# A DNA Binding Protein Is Required for Viral Replication and Transcription in *Bombyx mori* Nucleopolyhedrovirus

**DOI:** 10.1371/journal.pone.0159149

**Published:** 2016-07-14

**Authors:** Cui Zhao, Chen Zhang, Bin Chen, Yanghui Shi, Yanping Quan, Zuoming Nie, Yaozhou Zhang, Wei Yu

**Affiliations:** 1 College of life sciences, Zhejiang Sci-Tech University, Zhejiang Province, Hangzhou 310018, China; 2 Zhejiang Provincial Key Laboratory of Silkworm Bioreactor and Biomedicine, Zhejiang Province, Hangzhou 310018, China; Institute of Plant Physiology and Ecology, CHINA

## Abstract

A DNA-binding protein (DBP) [GenBank accession number: M63416] of *Bombyx mori* nuclear polyhedrosis virus (BmNPV) has been reported to be a regulatory factor in BmNPV, but its detailed functions remain unknown. In order to study the regulatory mechanism of DBP on viral proliferation, genome replication, and gene transcription, a BmNPV *dbp* gene knockout virus *dbp*-ko-Bacmid was generated by the means of Red recombination system. In addition, *dbp-*repaired virus *dbp*-re-Bacmid was constructed by the means of the Bac to Bac system. Then, the Bacmids were transfected into BmN cells. The results of this viral titer experiment revealed that the TCID_50_ of the *dbp*-ko-Bacmid was 0; however, the *dbp*-re-Bacmid was similar to the wtBacmid (*p>*0.05), indicating that the *dbp*-deficient would lead to failure in the assembly of virus particles. In the next step, Real-Time PCR was used to analyze the transcriptional phases of *dbp* gene in BmN cells, which had been infected with BmNPV. The results of the latter experiment revealed that the transcript of *dbp* gene was first detected at 3 h post-infection. Furthermore, the replication level of virus genome and the transcriptional level of virus early, late, and very late genes in BmN cells, which had been transfected with 3 kinds of Bacmids, were analyzed by Real-Time PCR. The demonstrating that the replication level of genome was lower than that of wtBacmid and *dbp*-re-Bacmid (*p<*0.01). The transcriptional level of *dbp*-ko-Bacmid early gene *lef-3*, *ie-1*, *dnapol*, late gene *vp39* and very late gene *p10* were statistically significantly lower than *dbp*-re-Bacmid and wtBacmid (*p*<0.01). The results presented are based on Western blot analysis, which indicated that the lack of *dbp* gene would lead to low expressions of *lef3*, *vp39*, and *p10*. In conclusion, *dbp* was not only essential for early viral replication, but also a viral gene that has a significant impact on transcription and expression during all periods of baculovirus life cycle.

## Introduction

The *Bombyx mori* nuclear polyhedrosis virus (BmNPV) is a typical member of the insect baculoviruses, a family of double-stranded DNA (dsDNA) viruses with large circular genomes. Although the genome of BmNPV is 5481bp shorter than that of *Autographa californica* multiple NPV (AcMNPV), there is a very close relationship between their genomes [1]. Thus, the potential protein coding regions, gene structure, viral DNA replication initiation site, and the existence of regulatory elements of BmNPV can be predicted by aligning with those of AcMNPV. There are 136 open reading frames (ORFs) in BmNPV, in which only a few had been identified and most of their functions were inferred from the corresponding AcMNPV genes [2, 3]. Most ORFs of the BmNPV are over 90% homologous with AcMNPV, but subtle changes often result in significant differences in morphology, infection dynamics, and host range [4–6].

In total, there are 65 amino acids that the 198 bp BmNPV DBP ORF; the predicted protein molecular weight is 8.08 KD and the isoelectric point is 12.46. According to hydrophobicity analysis by bioinformatics, the DBP shows strong hydrophilicity as a whole [7]. The DBP protein does not have a transmembrane region and signal peptide, indicating that it is not a transmembrane protein. The DBP protein is arginine-rich at its N-terminal, without the N-terminal closed or modified, such as glycosylation or phosphorylation. The amino acid sequence showed high homology to that of AcMNPV (97%) [7–9]; however, the BmNPV basic protein possessed an additional sequence of 10 amino acids that contains R-R-R-S-S in the BmNPV protein. The sequence of amino acids appears 3 times in the basic protein DBP of BmNPV, while appearing twice in the AcMNPV and OpNPV [10, 11]. The basic protein DBP is considered to be involved in the neutralization of viral DNA by arginine residues, and plays an important role in depolymerizing the virus through the phosphorylation of serine and threonine during the infection process [12].

Although our previous study demonstrated that DBP could interact with a polyhedron promoter to enhance the transcriptional activity of polyhedron promoter [13], the detailed functions that occur during the baculovirus life cycle remain unknown. Therefore, the *dbp* gene was knocked-out by Red recombination system and repaired by Bac to Bac system in this study, in order to study the overall role of BmNPV *dbp* during the infection process [14, 15]. After the transfection of these viruses into BmN cells, the replication of BmNPV genomic DNA, and the transcription levels of early, late, and very late genes were determined. This research lays the foundation for the in-depth understanding of the biological function of *dbp* in the BmNPV life cycle.

## Materials and Methods

### Materials

In our laboratory, we stored the following *Escherichia coli* strains: TG1, DH10Bac (containing helper plasmid), BW25113 (containing plasmid pKD46 and can express Red recombinase), plasmid pKD3 (containing the anti-chloromycetin gene *cat*), and pFastBac1. Liquid SOC medium was purchased from Biocolor BioScience & Technology Company (Shanghai, China); *L*- arabinose was purchased from Promega Co. (Madison, WI, USA). DNA ladder marker, T4 ligation, Taq enzyme, and restriction endonucleases were obtained from Takara Bio Inc. (Tokyo, Japan). KOD plus high-fidelity enzyme was purchased from TOYOBO Co., Ltd. (Ohtsu, Japan). Anti-His tag monoclonal antibody was purchased from Zexiyuan Biotechnology Company (Beijing, China). The monoclonal anti- rabbit antibodies *LEF-3*, *VP39*, and *P10* were designed and produced by Abmart Medicine Company (Shanghai, China). The specific primers were synthesized by Sangon Biotech (Shanghai) Co., Ltd. (Shanghai, China).

### Targeting linear fragment preparation

To produce a *dbp* gene-deficient bacmid, a targeting linear fragment of approximately 1100 bp was constructed by PCR using the pKD3 as the template and dbp-C1&dbp-C2 as the primers (Table 1). The dbp-C1 and dbp-C2 contain a 50 bp homologous arm of *dbp* (underlined) and the 20 bp *cat* homologous domain, respectively. In final, a “taa” box was introduced artificially as the termination codon. The amplification program was: one cycle of 94°C for 2 min, and 30 cycles of 94°C for 15 sec and 60°C for 30 sec, elongation at 72°C for 1 min and a final elongation step at 72°C for 10 min. The PCR products were separated by electrophoresis through 1% (w/v) agar gel. The linear DNA fragments were purified by the Gel Recovery Kit, then dissolved in 30 μL of ultrapure water and stored frozen at -20°C.

**Table 1:** Primer sequences for PCR amplification.

Primer name	Primer sequences (5'→3')
dbp-C1	Forword:5'-ATAAATTACACAATTTAAACATGGTTTATCGTCGCCGTCGT CGTTCTtaaGTGTAGGCTGGAGCTGCTTC-3
dbp-C2	Reverse:5'-CTGGATCTTCTGTATGTGCGAGGTCTACCCGGGCGGCGTC TGTAACCCGAATGGGAATTAGCCATGGTCC -3'
dbp-F	Forword:5’-GCCGTGTCCAATTGCAAGTTCAAC-3’
dbp-R	Reverse:5’-GCGGGCTTAGTTTAAAATCTTGCAGAC-3’
cat-F	Forword:5’-CACGTTTAAATCAAAACTGGTG-3’
cat-R	Reverse:5’-CAATATGGACAACTTCTTCG-5’
p1dbp-F	Forword:5’-CCGGAATTCACTATGCCGTGTCCAATT-3’ (the *Bam*H I restriction site is underlined)
p1dbp-R	Reverse:5’-CCGCTCGAGTTAATAGTAGCGTGTTCTGT-3’ (the *Xho* I restriction site is underlined)
M13-F	Forword:5’-GTTTTCCCAGTCACGAC-3’
M13-R	Reverse:5’-CAGGAAACAGCTATGAC-3’
lef-3-F	Forword:5’-TCGGATGACCGTTCTACCTCTT-3’
lef-3-R	Reverse:5’-CTTCCAGCAGCATTGAGATTTG-3’
ie-1-F	Forword:5’- CGAGACGGCTGCACAAAA -3’
ie-1-R	Reverse:5′-TGCCCAAAAGAAACCCACA-3′
dnapol-F	Forword:5′-GCCGATTTGCGTTTTTTGCC-3′
dnapol-R	Reverse:5′-GATTGCCATTTGTGCTTGTT-3′
vp39-F	Forword:5’-AGACACCACAAACCCGAACAC-3’
vp39-R	Reverse: 5’-TTGATCGCCAACACCACCT-3’
p10-F	Forword:5’-GACACGAATTTTAGACGCCATT-3’
p10-R	Reverse:5’-CGATTCTTCCAGCCCGTTT-3’
β-Actin-F	Forword:5’-GCGCGGCTACTCGTTCACTACC-3’
β-Actin-R	Reverse:5’-GCGCGGCTACTCGTTCACTACC-3’

These two primers were composed of about 50-bp *dbp* homology arms (underlined) and a 20-bp *cat* homologous zone. Letters in boxes represent the restriction enzymes *Bam*H I and *Eco*R I.

### Construction of the *dbp*-knockout and repaired bacmid

#### Construction of the *dbp* gene-knockout Bacmid

The constructed targeting linear fragment was transformed into DH10Bac cells. The expression of λ Red recombinase can be induced after *L*-arabinose is added. The recombinase induces homologous recombination between *dbp* and the targeting linear fragment [16]. The *dbp*-ko-Bacmid positive clones were selected by LB solid plates, which contained chloramphenicol and kanamycin (S1A–S1C Fig). Different PCR primer pairs were used to confirm whether *dbp* gene was knocked out and the 3 pairs of primers included dbp-F&dbp-R, dbp*-F*&cat-R, and cat-F&dbp-R (Table 1).

#### Construction of the *dbp* gene-repaired Bacmid

On the basis of the *dbp* sequence, a primer pairs were designed by DNAStar, and the *Eco*R I and *Xho* I restriction sites were introduced into the upstream and downstream primes, respectively. The primer pairs of *dbp* were p1dbp-F and p1dbp-R (Table 1) and the amplified sequence included 205 bp upstream of the *dbp* gene (contain *dbp* gene’s promoter sequence). In brief, p1dbp-F and p1dbp-R were used as the primes and wtBacmid as the template to amplify a sequence containing *dbp* gene promoter sequence and the corresponding ORF. Then, the purified PCR products and pFastbac1 plasmid were digested by *Bam*H I and *Eco*R I; then, the digested products were used to generate the recombinant plasmid pFastBac1-*dbp*. Next, the recombinant plasmid was transfected into DH10bac competent cells together with *dbp*-ko-Bacmid to obtain the repaired virus (S1D–S1F Fig).

M13 primers and *dbp* gene-specific primers were used for the PCR confirmation with the different combinations of primers included M13-F(-40)&M13-R, M13-F(-40) &p1dbp-R and p1dbp-F&M13-R (Table 1). The PCR reaction program was: one cycle of 95°C for 3 min; 30 cycles of 94°C for 45 sec and 55°C for 45 sec; elongation at 72°C for 5 min; and a final elongation step at 72°C for 10 min.

### Quantitative PCR analysis of viral DNA replication

BmN cells in the logarithmic growth phase (1×10^6^ cells/mL) were transfected with 1.0 μg DNA from the 3 kinds of Bacmids (*dbp*-ko-Bacmid, *dbp*-re-Bacmid and wtBacmid). Then, cells containing Bacmids were harvested at 6, 12, 24, 48, 60, and 72 h. After total DNA was extracted, *Dpn*Ⅰ was used to identify and enzymolyze methylation sites in order to remove the exogenous DNA introduced during transfection. Then, samples were analyzed by qPCR using the progeny viral genomic DNA as the template and gp41F&gp41R as the primers (Table 1) [17]. The qPCR amplification program was as follows: one cycle of 95°C°C for 10 min, 40 cycles of 95°C for 10 sec, 50°C for 10 sec, and elongation at 72°C for 12 sec. Dissolve curve program is: 95°C for 5 s, 65°C for 1 min, 95°C for 15 s. A relative quantitative method (△△*C*_*t*_) was used to evaluate the transcriptional levels.

### Virus titer detection

BmN cells in the logarithmic growth phase were transfected with the 3 kinds of Bacmids as described above. Then, viral supernatants of the Bacmids were collected after 6 h cultivation at 27°C and diluted from 10^−1^ to 10^−13^. BmN cells were seeded in 96-well plates at 1×10^3^ cells/well and each serial dilution was inoculated into the wells, using the normal BmN cells as control. Each dilution was repeated 8 times. The plates were incubated at 27°C for 5 to 7 days. The viral titer (TCID_50_) was calculated according to the method of Reed and Muench.

### Real-time RT-PCR analysis of the transcriptional phases of *dbp* gene

BmN cells in the logarithmic growth phase were infected with BmNPV, then cultivated at 27°C before the cells were harvested at 1.5, 3, 6, 9, 12, 24 and 48 h post-infection. Specific primers were designed on the basis of the sequences of viral early gene *lef3*, late gene *vp39*, very late gene *p10* and *dbp* gene from the BmNPV genome [18, 19]. The primer pairs lef-3-F and lef-3-R, vp39-F and vp39-R, p10-F and p10-R, and dbp-C1 and dbp-C2 were used (Table 1).

### Real-time RT-qPCR analysis of the viral gene transcription at different phases

To examine the effect of BmNPV *dbp* gene on viral gene transcription during the process of BmNPV infection, BmN cells were transfected with 2 μg liposome-encapsulated DNA of *dbp*-ko-Bacmid, *dbp*-re-Bacmid or wtBacmid in the logarithmic growth phase, respectively. Next, the cell culture supernatants were harvested at 12, 24, 48, and 72 h post-transfection. Total RNA was extracted with TRizol^®^ and digested with DNase I to remove any residual DNA. The total RNA was reverse transcripted into the first-strand of cDNA using the digested RNA as template and oligo-dT as primer [15]. Specific primers were designed on the basis of the sequences of viral early gene *lef3*, *ie-1*, *dnapol*, late gene *vp39* and very late gene *p10* from the BmNPV genome. The primer pairs which were lef-3-F and lef-3-R, ie-1-F and ie-1-R, dnapol-F and dnapol-R, vp39-F and vp39-R and p10-F and p10-R were used (Table 1). Bmβ-Actin was used as the internal reference and its primers sequence were β-Actin-F and β-Actin-F (Table 1).

### Western Blot analysis of the viral gene expression at different phases

BmN cells were transfected with *dbp*-ko-Bacmid, *dbp*-re-Bacmid or wtBacmid, respectively. After 48 h the cells were harvested and lysed with lysis solution (50 mM Tris–HCl pH 8.0, 150 mM NaCl, 5 mM EDTA, 1 mM DTT, 0.5% (v/v) NP-40, 0.5% (w/v) sodium deoxycholate containing the protease inhibitor phenylmethylsulfonyl fluoride (PMSF) at a final concentration of 100 μg/mL). Western blotting was conducted with 6×his-tag monoclonal antibody after the sample preparation. Furthermore, the 48 h post-transfected BmN cells (*dbp*-ko-Bacmid, *dbp*-re-Bacmid and wtBacmid) were collected and 10 μg proteins were subjected to SDS-PAGE, and then incubated with a series of monoclonal antibodies LEF-3, VP39, and P10 for identification using Western blotting.

### Electron microscopy

BmN cells transfected with *dbp*-ko-Bacmid, *dbp*-re-Bacmid or wtBacmid were harvested and washed with PBS (pH 7.4) after 24 p.t., then the cells were fixed with 2.5% glutaraldehyde in phosphate buffer (pH7.0) overnight at 4°C; washed and post-fixed with 1% OsO_4_ in phosphate buffer (pH7.0) for 1–2 h; then the cells were washed again and dehydrated by a graded series of ethanol (30%, 50%, 70%, 80%, 90%, 95%) for approximately 15 min at each step. After being transferred to absolute ethanol for 20 minutes, the specimen was placed in 1:1 mixture of absolute acetone and the mixture was embedded in medium for 1 h at room temperature, then transferred to 1:3 mixture of absolute acetone and embedding medium mixture for 3 h and to absolute embedding medium for more than 12 h. After being heated at 70°C overnight, the specimen was placed into Reichert microtome to be sliced into sections from 70–90 nm and stained by uranyl acetate and alkaline lead citrate for 15 minutes, respectively, the specimen sections were observed under TEM of HitachiH-7650.

## Results

### Confirmation of *dbp*-knockout and repaired bacmids

The recombinant plasmid was constructed by replacement with a targeted linear fragment. Single colonies were randomly selected from agar plate and cultured overnight in liquid LB medium containing chloromycetin and kanamycin. *dbp*-ko-bacmid DNA was confirmed with PCR using wtBacmid as the control and the following primer pairs: dbp-F and dbp-R, dbp-F and cat-R and cat-F and dbp-R. The theoretical lengths of the amplified products should be about 660 bp, 990 bp and 1600 bp, while the lengths of the control group were to be 516 bp, 0 bp, and 0 bp (S2A Fig).

Bac to Bac system was used to construct the *dbp* gene repaired Bacmid. Positive single colonies were randomly selected from agar plates containing gentamicin, kanamycin, tetracycline, IPTG and X-gal, and cultured overnight in liquid LB medium containing gentamicin, kanamycin, and tetracycline. The theoretical lengths of the amplified products were projected to be 3200 bp, 2600 bp, and 1450 bp, respectively; primer pairs were M13-F(-40)&M13-R, M13-F(-40) &p1dbp-R and p1dbp-F&M13-R (S2B Fig).

### Effect of *dbp-*knockout on viral genome replication

**Fig 1:**
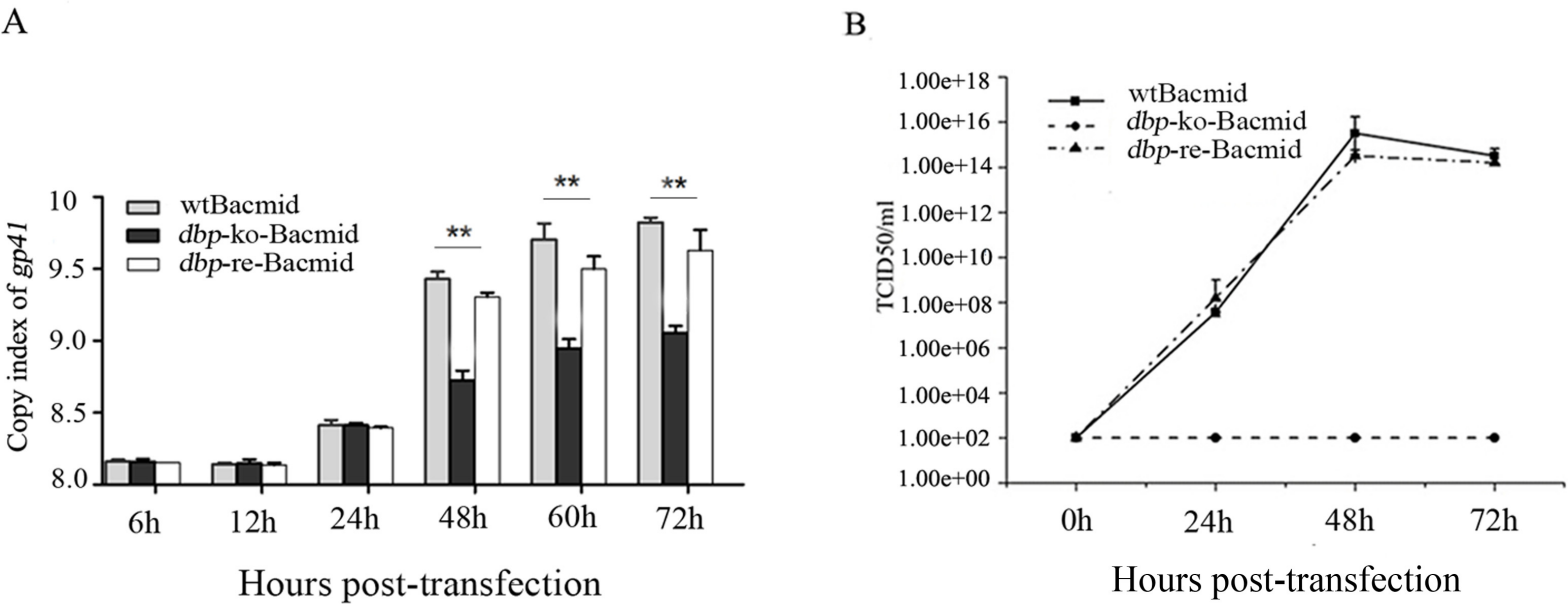
Effect of *dbp-*knockout on viral replication. (A) BmN cells transfected with *dbp*-ko-Bacmid, *dbp*-re-Bacmid or wtBacmid were harvested at 6–72 h p.t. and analyzed for *gp41* by RT-qPCR for the viral genome copy number. (B) The BmN cells were transfected with the Bacmids, harvested at 0~72 h p.t., and analyzed for the virus titers. **p<0.01 *vs* wtBacmid and *dbp*-re-Bacmid. Values are expressed as means±SEM. Similar results were obtained in three independent experiments.

After quantitative PCR analysis, the results showed that the genomic DNA copies of 3 kinds of Bacmids (*dbp*-ko-Bacmid, *dbp*-re-Bacmid and wtBacmid) did not show obvious differences within 24 h (*p*>0.05), while during the 48–72 h, the copies had risen in varying degrees. The DNA copies of wtBacmid and *dbp*-re-Bacmid were significantly higher than that of *dbp*-ko-Bacmid (Fig 1A) (*p*<0.01).

The *dbp*-ko-Bacmid, wtBacmid, and *dbp*-re-Bacmid were harvested from the supernatants of the BmN cells. The results from virus titers detection showed that *dbp*-ko-Bacmid lost the ability to generate budded virus (BV), but the *dbp*-re-Bacmid could recover the ability of progeny production and the titers of BV generated by wtBacmid and *dbp*-re-Bacmid were similar, suggesting that *dbp* gene product was essential for BV assemble (Fig 1B).

### The transcriptional phases of *dbp* gene

**Fig 2:**
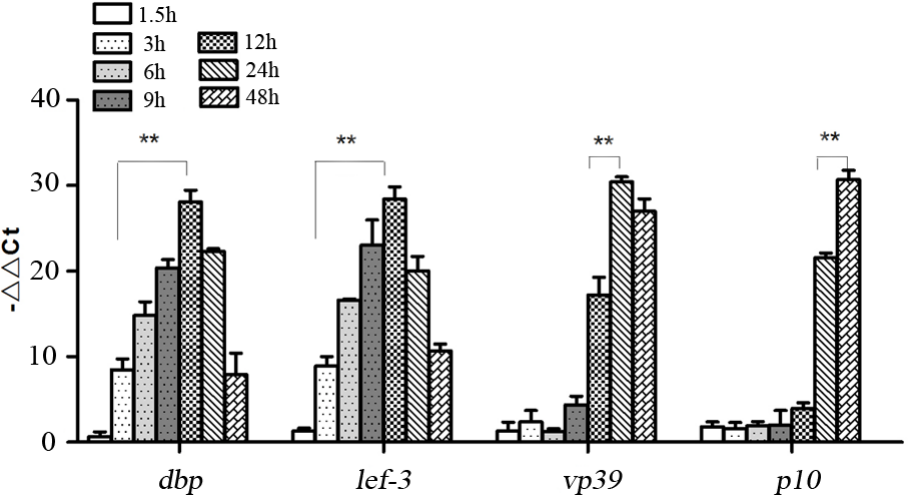
RT-qPCR analysis of the transcriptional phases of *dbp* gene in cells infected with wtBacmid. **P <0.01 the maximum transcription value *vs* the first detected value of *dbp*, *lef-3*, *vp39*, and *p10*.

The transcriptional phases of *dbp* gene in BmN cells, which had been infected with BmNPV, were analyzed by RT-qPCR. The results of qPCR of *dbp* gene showed that the transcript of *dbp* gene was first detected at 3 h post-infection, indicating that *dbp* gene is an early gene of BmNPV to be transfected. In detail, the maximum value was detected at 12 h post-infection, which was significantly higher than that of 3 h (*p*<0.01). Furthermore, the transcript of *lef-3*, *vp39*, and *p10* were first detected at 3 h, 12 h, and 24 h, respectively, which consistent with the theoretical values. Moreover, the maximum value of the results of qPCR about *lef-3*, *vp39*, and *p10* were detected at 12 h, 24 h, and 48 h post-infection, respectively, which were all significantly higher than that of the first detected phase (*p*<0.01) (Fig 2).

### Effects of *dbp*-knockout on BmNPV gene transcription

**Fig 3:**
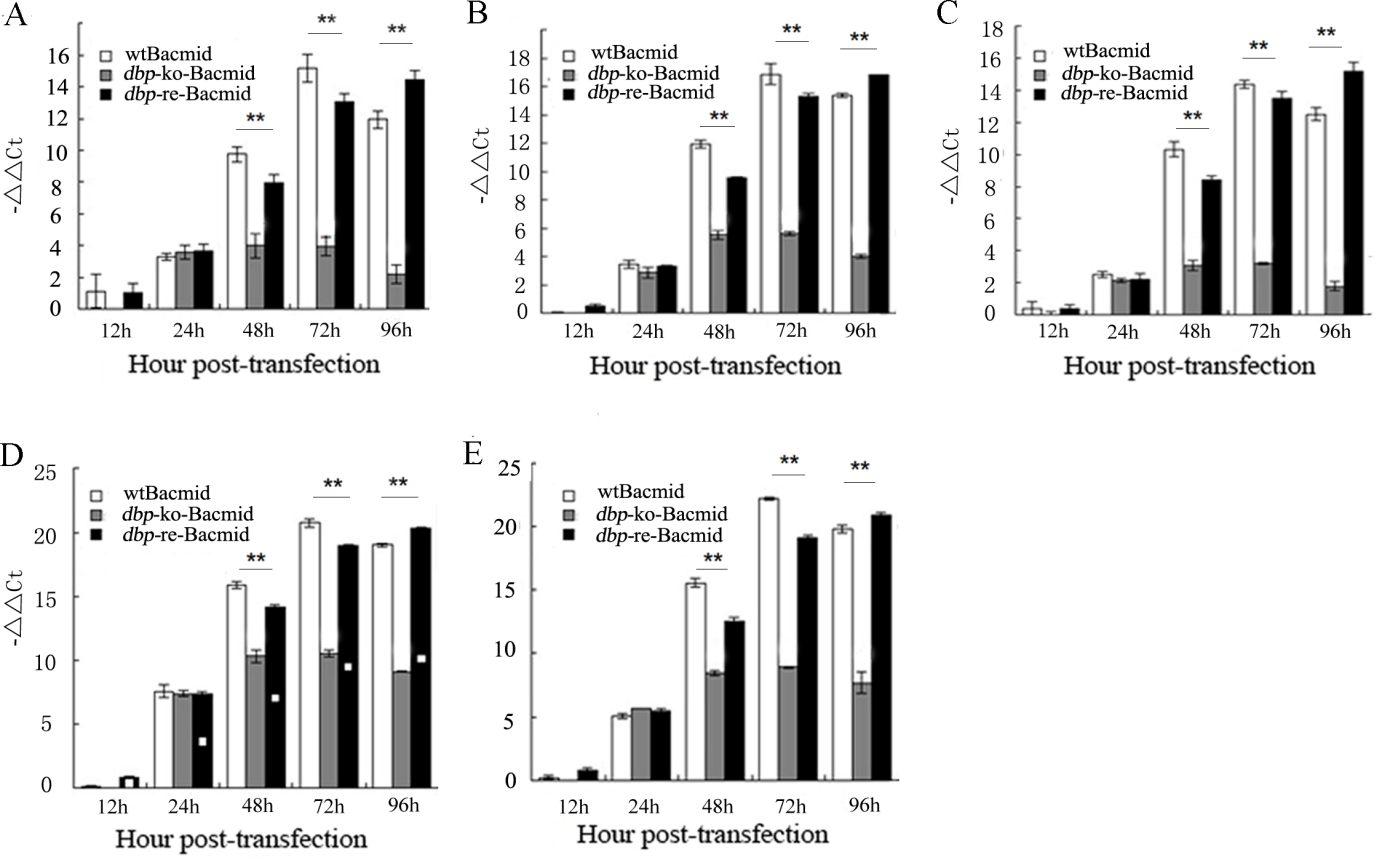
RT-qPCR analysis of the transcription levels of viral genes in cells transfected with Bacmids. (A-C) early gene *lef-3*, *ie-1* and *dnapol*. (D) late gene *vp39*. (E) very late gene *p10*. **p<0.01 *vs* wtBacmid and *dbp*-re-Bacmid.

The gene expression in BmNPV is mainly regulated at the transcriptional level. Early gene, late gene, and very late gene from the 3 kinds of Bacmids were analyzed by RT-qPCR [16]. To determine the effects of *dbp*-knockout on BmNPV gene transcription, several early genes were analyzed that included *lef-3*, *ie-1*, and *dnapol*. The results determined that there was little difference among *dbp*-ko-Bacmid, wtBacmid, and *dbp*-re-Bacmid during 12–24 h post-transfection. However, *dbp*-re-Bacmid and wtBacmid were significantly higher than *dbp*-ko-Bacmid at 48, 72, and 96 h post-transfection (*p*<0.01). In detail, the transcription levels of 3 early genes (*lef-3*, *ie-1*, *dnapol*) of *dbp*-ko-Bacmid changed little after 24 h post-transfection (Fig 3A–3C). As to the late gene *vp39*, the transcription level of *dbp*-ko-Bacmid, wtBacmid, or *dbp*-re-Bacmid also stayed at the same within 24 h post transfection (p.t.). While the transcription levels of *dbp*-re-Bacmid and wtBacmid were much higher than that of *dbp*-ko-Bacmid at 48, 72, and 96 h p.t. (Fig 3D). Similarly, to the very late gene *p10*, the transcription level of *dbp*-ko-Bacmid, wtBacmid, or *dbp*-re-Bacmid stayed at the same levels during 12–24 h p.t. While the transcription of *dbp*-re-Bacmid and wtBacmid were much higher than that of *dbp*-ko-Bacmid after 24 h post-transfection (*p*<0.01). However, the *dbp*-ko-Bacmid changed little after 24 h (Fig 3E).

### Effects of *dbp*-knockout on viral genes expression

**Fig 4:**
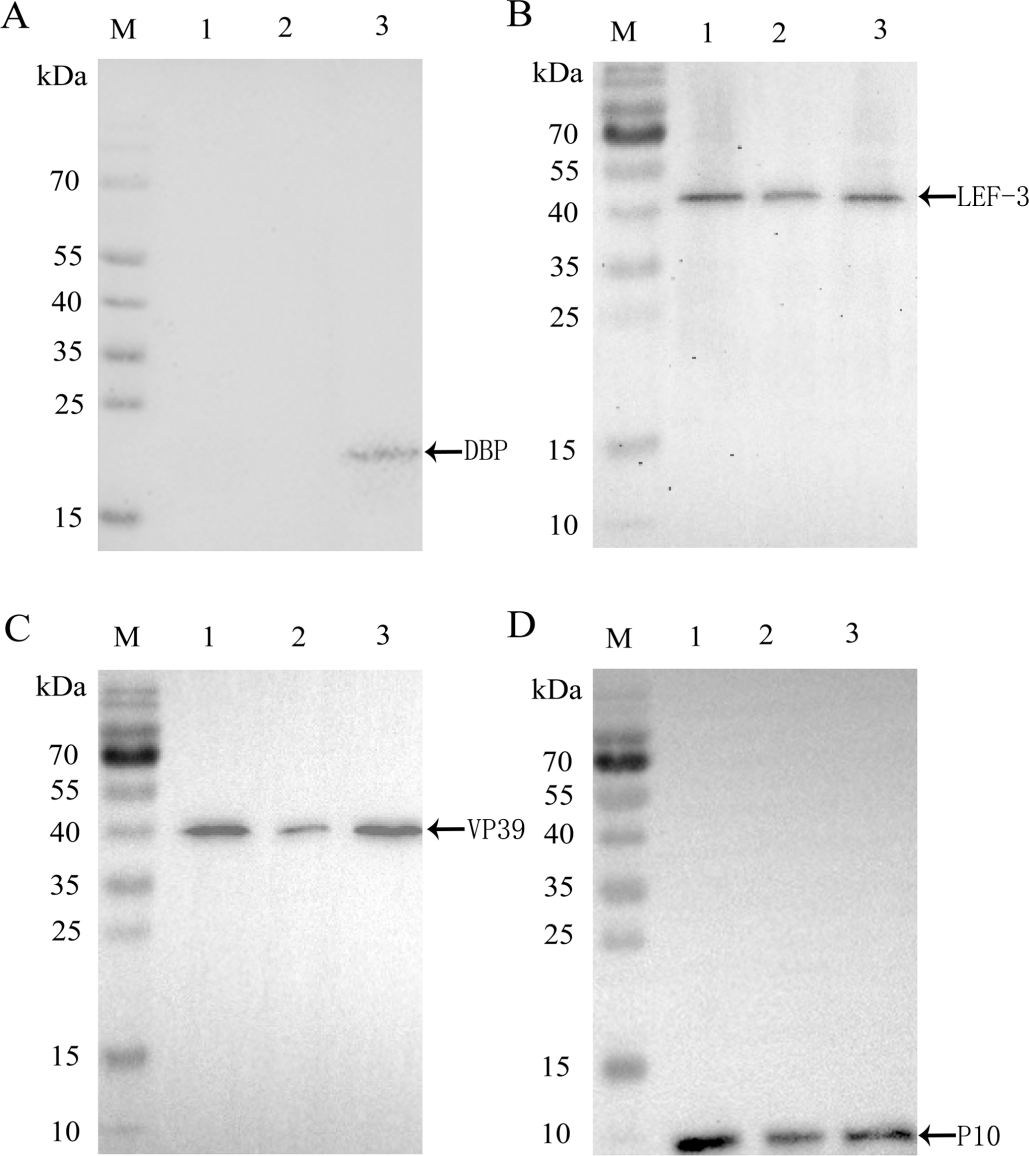
Western blot analysis of DBP and expression levels of viral proteins in BmN cells transfected with Bacmids. (A-D) BmN cells transfected with *dbp*-ko-Bacmid, *dbp*-re-Bacmid, or wtBacmid were harvested at 48 h p.t. and analyzed by 6×his-tag monoclonal antibody or series of monoclonal antibodies DBP, LEF-3, VP39 and P10, respectively. Lanes M: protein markers; lane1: wtBacmid; lane 2: *dbp*-ko-Bacmid; lane 3: *dbp*-re-Bacmid.

The cell lysates of the BmN cells transfected with *dbp*-ko-Bacmid, wtBacmid, and *dbp*-re-Bacmid at 48 h p.t. were collected for Western blot analysis. The results showed that (Fig 4A) *dbp*-re-Bacmid was constructed successfully. Then, BmN cells were transfected with *dbp*-ko-Bacmid, wtBacmid, or *dbp*-re-Bacmid and harvested at 48 h p.t. The resulting cell lysates were collected for Western blot analysis with LEF-3, VP39, and P10 monoclonal antibodies. Cells transfected with *dbp*-ko-Bacmid had no obvious hybridization band, while cells transfected with *dbp*-re-Bacmid and wtBacmid had an obvious hybridization band at 45 kDa, 40 kDa and 10 kDa, respectively. The results showed that (Fig 4B–4D) knockout *dbp* gene significantly affected viral gene transcription and expression in all periods of the cell life cycle.

### Results of observation under transmission electron microscopy

**Fig 5:**
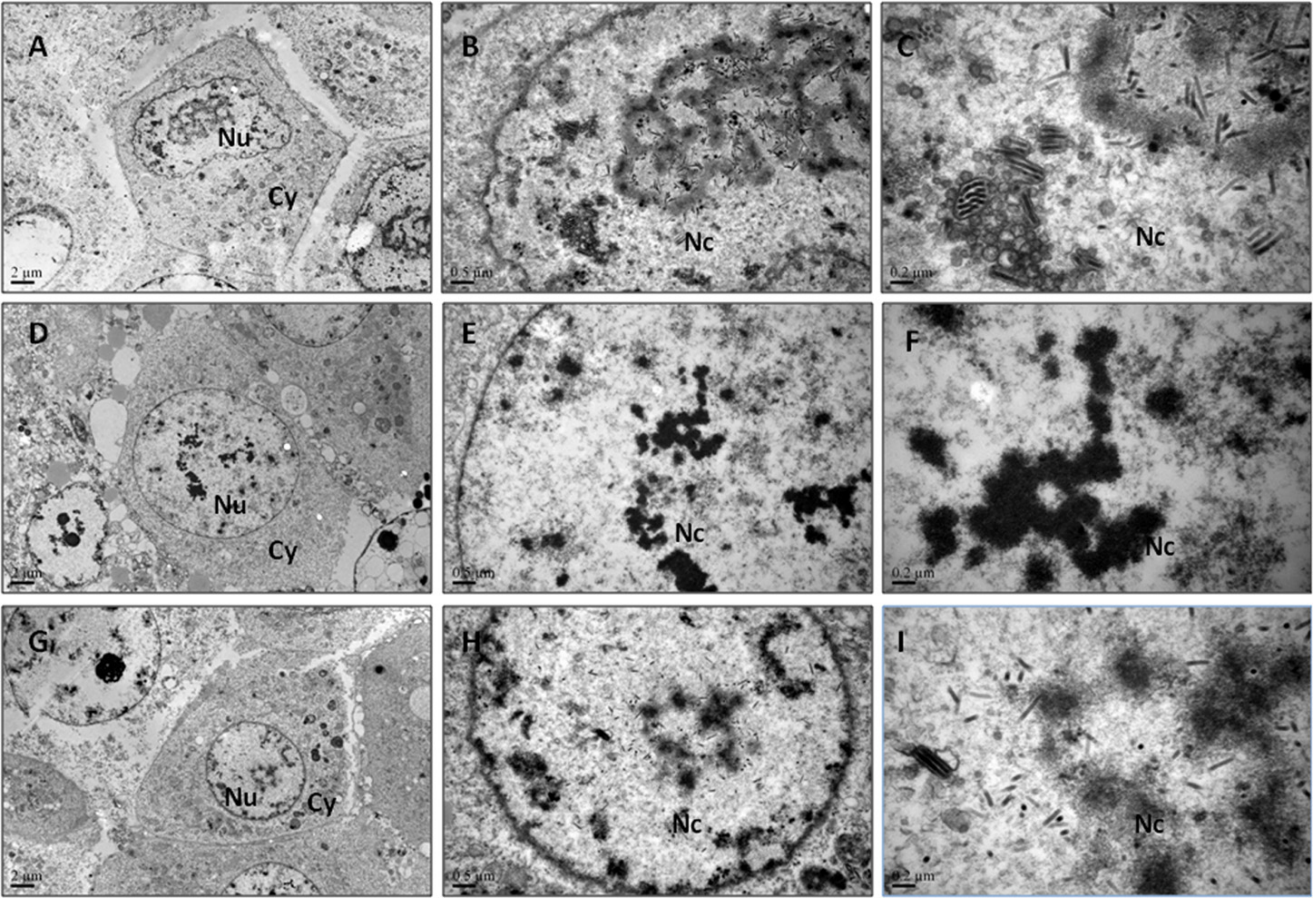
Electron microscopic analysis of cells transfected with the bacmids. (A-C) BmN cells were analyzed at 24 h p.t. with wtBacmid: 5000×, 20000× and 50000×. (D-F) BmN cells were analyzed at 24 h p.t. with *dbp*-ko-Bacmid: 5000×, 20000× and 50000×. (G-I) BmN cells were analyzed at 24 h p.t. with *dbp*-re-Bacmid: 5000×, 20000× and 50000×. Nu, nucleus; Cy, cytoplasm; Nc, nucleocapsid. Bars indicate the scale.

In order to analyze the impact of *dbp* deletion on virus particle assemble, the sections of BmN cells transfected with 3 kinds of Bacmids were observed under the transmission electron microscopy. The viral particles were observed under 5000×, 20000×, and 50000× at 24 h post-transfection with wtBacmid (Fig 5A–5C), *dbp*-ko-Bacmid (Fig 5D–5F) or *dbp*-re-Bacmid (Fig 5G–5I). There were no BmNPV particles found when observed after 24 h p.t. with *dbp*-ko-Bacmid (Fig 5D–5F). In contrast, BmNPV particles were viewed again after the cells transfected with repaired virus *dbp*-re-Bacmid (Fig 5G–5I). These results showed clearly that *dbp* had an affect on virus assembly.

## Discussion

The *dbp* is a conserved gene that is widely present in the baculovirus genome and is considered to be a core gene of the virus. Although our previous work has shown that it is possible to affect the transcription of BmNPV in very late genes [13], the detailed function of *dbp* in the baculovirus life cycle remains largely unknown.

In order to investigate the biological function of *dbp* on the replication and transcription of BmNPV, a *dbp* gene knockout virus *dbp*-ko-bacmid was constructed by the Red recombination system. The traditional way to knock out a gene from the baculovirus genome is to co-transfect the cells with transfer vectors and wild type bacmid; then, the recombinant viruses are obtained by homologous recombination and plaque screening [20, 21]. This process is time-consuming, laborious, and has low recombination efficiency. In the alternative, using the Red recombination system to knockout the targeted gene is high in recombination efficiency and quite convenience [22, 23]. Moreover, the Bac-to-Bac Baculovirus expression system was used towards the successful generation of a *dbp*-repaired virus, *dbp*-re-Bacmid [16]. The designed recombinant fragment included 205 bp upstream of the *dbp* gene, which contained the promoter sequence of the *dbp* gene. This was to ascertain that *dbp* gene expression had optimal timing.

The effects of *dbp*-knockout on viral DNA replication and gene transcription were further analyzed by RT-qPCR. First, the value-added viral titer detection results demonstrated that infectious budded virus (BV) was not generated when transfected by *dbp*-ko-bacmid, indicating that *dbp* gene was essential for the generation of progeny virus. In addition, the results showed that the genomic DNA copies of 3 kinds of Bacmids (*dbp*-ko-Bacmid, *dbp*-re-Bacmid and wtBacmid) did not show obvious differences within 24 h post-transfection. The results of the qPCR indicated that knockout *dbp* gene can inhibit the assembly of BV, but had no significant effect on viral genome replication. However, we also found that although the copies of *dbp*- ko-Bacmid were significantly decreased when compared with that of wtBacmid and *dbp*-re-Bacmid after 24 h post-transfection, the DNA copies of *dbp*-ko-Bacmid were steadily increasing. These results may have been caused by the failure of assembling virus particles of *dbp*-ko-Bacmid. The virus particles, which were released from the BmN cells infected with wtBacmid or *dbp*-re-Bacmid, can infect the cells again after 24 h post-infection.

These results indicated that the deletion of the *dbp* gene did not block the replication of virus genome entirely, but most likely was involved in the process of viral assemble and subsequent blockade of the budded virus (BV) production [16, 17, 24]. Electron microscopy further proved that infectious virus particles were not formed with *dbp*-ko-Bacmid.

Many previous studies have proved that knockout certain gene from BmNPV genome affects the transcription of virus genome [17, 25]. For example, Xing *et al*. constructed a Bm*P95* deletion virus that led to a defect in production of infections BV [17]. In our study, we also found that *dbp* deletion affected the transcription levels of viral early genes (*lef-3*, *ie-1*, *dnapol*), late gene (*vp39*), and very late gene (*p10*). A similar phenomenon was observed by Li *et al*., who revealed that AcMNPV P6.9, a homologous gene of *dbp*, is a precondition for the maximal hyper-expression of baculovirus’ very late genes [26].

Western blot results showed that the theoretical value size stripes of LEF-3, VP39, or P10 could only be observed in BmN cells transfected with *dbp*-re-Bacmid or wtBacmid, when incubated with 3 kinds of monoclonal antibodies. These results indicated that *dbp* is essential for the viral gene translation at different phases. These results may have been caused by a low concentration of virus particles or the failure of protein expression.

In summary, we speculate that the decreased levels of viral gene replication and transcription resulting from *dbp* deletion led to the failure of progeny virus production to assemble. As a type of BmNPV DNA binding protein, further in-depth study is required to clarify the mechanism of interaction between DNA and DBP.

## Supporting Information

S1 Fig Construction of *dbp* deficient and repaired bacmids. (TIF)

S2 Fig Validation of *dbp* deficient (A) and repaired bacmids (B) by PCR.
(TIF)

## Abbreviations

AcMNPV: *Autographa californica* multiple nuclear polyhedrosis virus
BmNPV: *Bombyx mori* nuclear polyhedrosis virus
BmN: *Bombyx mori* cells
DBP: DNA-binding protein
BV: budded virus
p.t.: post transfection
Lef: late expression factor
ORFs: open reading frames
