# Supplementary figures and images for: A DNA Binding Protein Is Required for Viral Replication and Transcription in Bombyx mori Nucleopolyhedrovirus
Source: PLoS One. 2016 Jul 14;11(7):e0159149. doi: 10.1371/journal.pone.0159149 (PMC4945074; doi:10.1371/journal.pone.0159149)

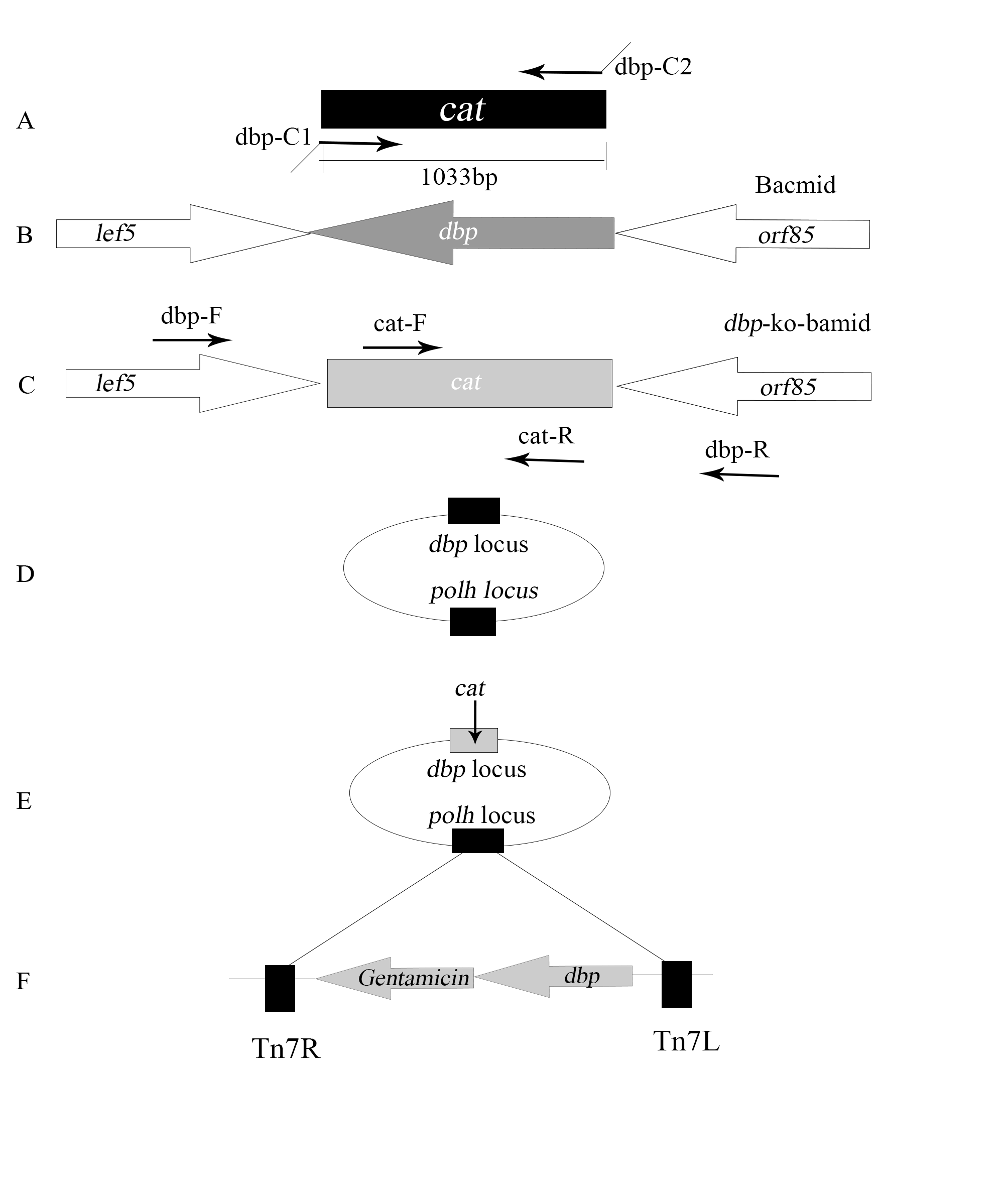

Supplement: S1 Fig — (TIF) [file pone.0159149.s001.tif]

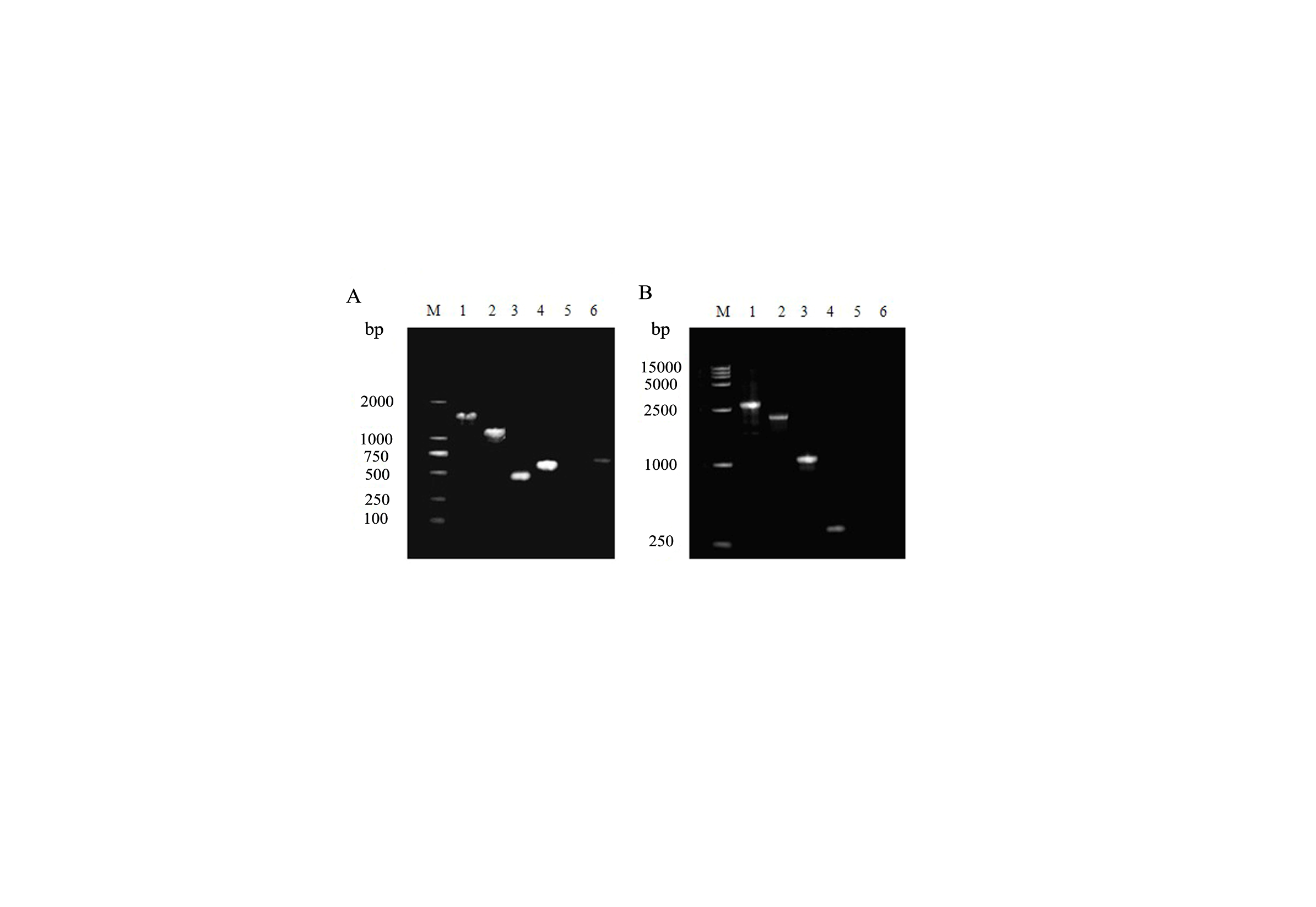

Supplement: S2 Fig — Validation of dbp deficient (A) and repaired bacmids (B) by PCR. (TIF) [file pone.0159149.s002.tif]
